# High throughput SNP discovery and genotyping in hexaploid wheat

**DOI:** 10.1371/journal.pone.0186329

**Published:** 2018-01-02

**Authors:** Hélène Rimbert, Benoît Darrier, Julien Navarro, Jonathan Kitt, Frédéric Choulet, Magalie Leveugle, Jorge Duarte, Nathalie Rivière, Kellye Eversole, on behalf of The International Wheat Genome Sequencing Consortium, Jacques Le Gouis, on behalf The BreedWheat Consortium, Alessandro Davassi, François Balfourier, Marie-Christine Le Paslier, Aurélie Berard, Dominique Brunel, Catherine Feuillet, Charles Poncet, Pierre Sourdille, Etienne Paux

**Affiliations:** 1 GDEC, INRA, Université Clermont Auvergne, Clermont-Ferrand, France; 2 Biogemma, Chappes, France; 3 IWGSC, Eversole Associates, Bethesda, Maryland, United States of America; 4 BreedWheat, Clermont-Ferrand, France; 5 Affymetrix, High Wycombe, United Kingdom; 6 EPGV US 1279, INRA, CEA, IG-CNG, Université Paris-Saclay, Evry, France; Institute of Genetics and Developmental Biology Chinese Academy of Sciences, CHINA

## Abstract

Because of their abundance and their amenability to high-throughput genotyping techniques, Single Nucleotide Polymorphisms (SNPs) are powerful tools for efficient genetics and genomics studies, including characterization of genetic resources, genome-wide association studies and genomic selection. In wheat, most of the previous SNP discovery initiatives targeted the coding fraction, leaving almost 98% of the wheat genome largely unexploited. Here we report on the use of whole-genome resequencing data from eight wheat lines to mine for SNPs in the genic, the repetitive and non-repetitive intergenic fractions of the wheat genome. Eventually, we identified 3.3 million SNPs, 49% being located on the B-genome, 41% on the A-genome and 10% on the D-genome. We also describe the development of the TaBW280K high-throughput genotyping array containing 280,226 SNPs. Performance of this chip was examined by genotyping a set of 96 wheat accessions representing the worldwide diversity. Sixty-nine percent of the SNPs can be efficiently scored, half of them showing a diploid-like clustering. The TaBW280K was proven to be a very efficient tool for diversity analyses, as well as for breeding as it can discriminate between closely related elite varieties. Finally, the TaBW280K array was used to genotype a population derived from a cross between Chinese Spring and Renan, leading to the construction a dense genetic map comprising 83,721 markers. The results described here will provide the wheat community with powerful tools for both basic and applied research.

## Introduction

Because they are the most abundant type of polymorphism in plant and animal genomes and because they are amenable to high-throughput, cost effective genotyping technologies, Single Nucleotide Polymorphisms (SNPs) have been adopted as the markers of choice in genetics. In the past years, they have been widely used for various applications, including genome-wide association studies, characterization of genetic resources, marker-assisted breeding and genomic selection [1,2]. The power of these different approaches relies heavily on marker density and on the ability to assay thousands of SNPs in parallel. Compared to other types of markers such SSRs or Diversity Array Technology (DArT) markers, SNP discovery relies on the comparison of homologous sequences between genotypes to identify variations at the sequence level [3]. If the advent of next generation sequencing systems opened the way to whole genome resequencing of several small to medium genome plant species for SNP discovery [4–8], the size of the wheat genome has long hampered such approaches. As a result, most of the SNP discovery initiatives that have been conducted to date relied on complexity reduction approaches. For example, Winfield *et al*. [9] used an exome capture array to target ~57 Mb of coding sequences in 43 bread wheat accessions and wheat relatives and discovered 921,705 putative varietal SNPs. Similarly, a wheat exome capture targeting 107 Mb of non-redundant genic regions was used by Jordan *et al*. [10] to mine for SNPs in a panel of 62 wheat lines. Eventually, ~1.57 million SNPs were identified. In 2014, RNA-seq on a set of 19 bread wheat accessions led to the discovery of 67,686 variants [11]. Also, genotyping-by-sequencing has been applied to wheat [12]. Despite the fact that this technique has the potential to sample a higher fraction of the genome and especially intergenic regions that are not targeted by exome capture, the large amount of missing data limits SNP discovery. Recently, this approach was used by Jordan *et al*. [10] on a set of 62 diverse hexaploid lines and Kobayashi *et al*. [13] on the NIAS Japanese Wheat Core Collection consisting of 96 lines. In both studies, the number of SNPs was in the range of 200,000 to 300,000. Noticeable exceptions to complexity reduction approaches are the works conducted by Lai *et al*. [14] and Montenegro *et al*. [15] who used whole-genome resequencing data from 16 and 18 wheat accessions, respectively, to detect more than four million and 36.4 million SNPs on group 7 chromosomes and at the whole genome level, respectively. However, these two studies included mainly cultivars from Australia, leaving a significant part of the genetic diversity unexplored.

Concomitantly to SNP discovery, several technologies have been implemented for SNP genotyping, from low-throughput monoplex to ultra-high-throughput highly-multiplexed assays [16–20]. In 2013, Cavanagh *et al*. [21] developed an Illumina iSelect array containing ~9,000 gene-associated SNPs and used it to genotype 2,994 hexaploid wheat accessions. The year after, the same technology was used to design a 90K SNP array and characterize the wheat diversity in a set of almost 2,500 accessions [11]. In 2015, Winfield *et al*. [9] reported on the development of an Affymetrix Axiom 820K SNP array and its utilization to genotype 475 hexaploid wheat and wheat relative accessions. Recently, this array was used to design a breeder-oriented Axiom 35K SNP [22]. All these high-throughput arrays contain mainly gene-derived SNPs. However, bread wheat being an allohexaploid species, genes are present in multiple copies. In addition, the wheat genome has undergone more interchromosomal duplications than related grasses, with 20 to 30% of genes being duplicated [23–25]. As a result, genotyping is complicated by the presence of homoeologous and paralogous loci [11,16,21].

So far, the intergenic fraction of the wheat genome has been poorly exploited for SNP discovery. Nevertheless, while genes account for 1–2% of the genome, non-coding regions represent 98–99%, with 10–15% being low-copy sequences. Also, the repetitive fraction has been shown to be a great source of SNPs through the use of Insertion Site-Based Polymorphism (ISBP) markers [18,20]. Here we report on the exploitation of the whole wheat genome sequence for SNP development and genotyping. Using whole-genome resequencing data from eight wheat accessions, we discovered more than three million genome-wide SNPs and mined for single-copy loci to design a high-throughput genotyping array containing 280,226 SNPs. Out of them, 68.5% were converted from *in silico* putative SNPs to functional SNP assays, with almost one half producing diploid-like clusters, therefore demonstrating the efficacy of our design strategy. We also present the characterization of a 96-accession panel, as well as the construction of a dense genetic map comprising more than 83,000 loci.

## Material and methods

### Sequence data

Four European elite lines, namely Premio, Renan, Robigus and Xi19, were fully resequenced using Illumina HiSeq2000 sequencer with 2 x 100-bp paired end reads with a 500-bp insert size (study accession PRJEB16737).

Whole-genome Illumina paired-end resequencing data were retrieved from Bioplatforms Australia (http://www.bioplatforms.com/wheat-sequencing/) from two Australian (Westonia and Volcani), one Chinese (Xiaoyan54) and one Israeli (Yitpi) wheat lines. The read number and subsequent approximate sequencing depth varied between lines: 3,196,318,742 for Renan (18-fold coverage), 3,674,485,632 for Premio (16x), 1,902,221,314 for Robigus (11x), 3,218,293,718 for Xi19 (19x), 2,041,226,160 for Volcani (12x), 1,424,795,776 for Westonia (8x), 2,436,715,062 for Xiaoyan54 (14x) and 2,222,884,584 for Yitpi (13x).

Contigs from the chromosome-based draft sequence of the wheat genome [25] were used as a reference for read mapping. This set comprises 12,087,812 chromosome shotgun sequence (CSS) contigs longer than 200-bp representing 10,363,698,897 bp.

### Repeat detection and ISBP design

CSS contigs were analyzed using RepeatMasker (http://www.repeatmasker.org) with the TREP Plus library [26,27]. RepeatMasker results were processed with the IsbpFinder program to detect ISBP markers [20,28]. High and medium confidence ISBPs were filtered out through clustering to discard repeated sequences.

### Read mapping

Repeat-masked CSS contigs and ISBP sequences were indexed using the *index* function of the BWA software (version 0.6.1-r104) [29]. Raw reads of the eight cultivars were mapped without prior quality trimming using the BWA *aln* function with no mismatch allowed in the seed (k = 0), and a maximum of two mismatches in the read (n = 2). For CSS contigs, alignments were reported in the BAM/SAM format using BWA *sampe*. Mapped reads were filtered using Samtools view [30]. Only reads that were mapped in a properly paired mapped reads (f = 2) with a mapping quality greater than 20 (q = 20) were kept for further analysis. For ISBPs, BWA *samse* was used. BWA alignment files were merged and sorted using Samtools and awk command lines.

### SNP discovery

Samtools mpileup was used to convert BAM alignment format into BCF format, with parameters -u to generate BCF output, -E for extended Base Alignement Quality for higher sensitivity in local realignment around short indels, -D to output per-sample depth in BCF, -I to avoid indel calling and -f the path to the reference sequence file used for mapping. BcfTools was used for SNP calling, with options -c to make the call, -v to output potential variants positions only, -g to call genotypes at variants sites, -e to make likelihood-based analysis and -N to skip sites where the reference base is "N". Finally, vcfutils.pl varFilter was used to filter out potential SNPs with a minimum depth of coverage of 5 (-d), and a minimum Root Mean Square (RMS) mapping quality of 30. SNPs were classified into four different classes. These classes were based on the homozygous *vs*. heterozygous nature of the SNP in the selected panel: (1) the locus showed only homozygous AA and BB alleles among all ten lines, (2) the locus showed AA and BB and heterozygous AB alleles, (3) the locus showed only AA and AB alleles and (4) the locus was heterozygous AB in all ten lines. Only class 1 and class 2 SNPs were used in further analyses.

### Axiom genotyping

A list of SNPs was submitted to Affymetrix for probeset design. For each SNP, one single probeset was included in the TaBW280K SNP array. Genotyping was conducted on the Affymetrix GeneTitan system according to the procedure described by Affymetrix (Axiom^®^ 2.0 Assay Manual Workflow User Guide Rev3). Allele calling was carried out using a modified version of the Affymetrix proprietary software packages Affymetrix Power Tools (APT) and SNPolisher^™^ (http://www.affymetrix.com/estore/partners_programs/programs/developer/tools/devnettools.affx) to take into account the specificities of the wheat genome. For all SNPs, HomRO and HomFLD were calculated (http://media.affymetrix.com/support/developer/downloads/Tools/SNPolisher_User_Guide.pdf). The HomFLD filter was set to 3.6. As a first step, all the probesets were processed with a mild inbred penalty equal to 4 on all the samples. As a second step, the SNPs failing the QC criteria (“Other” and “NoMinorHom”) were reprocessed using an inbred penalty of 16. Probesets classified as OTVs by SNPolisher were analyzed with OTV_caller in the two steps. The TaBW280K SNP array can be purchased from Affymetrix.

### Variability estimation and diversity analysis

Variability for each locus was measured using the Polymorphism Index Content (PIC) (Anderson *et al*., 1993):

$$PIC\;=\;1-\sum_{i}^{n}p_{i}^{2}$$

where *p*i is the frequency of the *i*th allele. Genotyping data were used to describe diversity within the wheat panel. A dissimilarity matrix was built using simple matching coefficient between each pair of accessions and the diversity was analyzed by a Ward dendrogram and a Neighbor-Joining Tree. Data analyses were conducted using the DARwin software (http://darwin.cirad.fr/darwin) [31].

### Genetic mapping

PHR SNPs in the Chinese Spring x Renan F6 population were selected and filtered out to select diploidized probesets. A second filtration was applied to discarded markers which significantly (P ≤ 0.01) deviated from the expected 1:1 ratio in a chi-square test, markers with missing or heterozygous data in parents, markers with more than 15% missing data. SNPs were divided then into 21 different sets corresponding to the 21 chromosomes, based on their CSS-based *in silico* assignment. Genetic maps were then constructed using MSTmap [32] with the following default parameters: population type: RIL6; distance function: Kosambi; cut-off: 0.00000000001; map dist.: 15; map size: 2; missing threshold: 0.20; estimation before clustering: yes; detect bad data: yes; objective function: ML. Once robust framework maps were obtained for each chromosome, a whole-genome map was built using a set of 5,230 selected markers covering all chromosomes and genetic bins. This map was used to place additional markers consisting in unassigned markers from previous chromosome-per-chromosome analyses, as well as markers that were excluded during the second filtration phase.

## Results

### De novo SNP discovery in the hexaploid wheat genome

Whole genome resequencing data from four European wheat cultivars (Premio, Renan, Robigus and Xi19), as well as from two Australian (Westonia and Yitpi), one Chinese (Xiaoyan54) and one Israeli (Volcani) cultivars were used to mine for SNPs in the hexaploid wheat genome. To this aim, reads were mapped to the chromosome-based draft genome sequence [25]. For SNPs located in low-copy regions, the repeat-masked contigs were used as a reference. This dataset comprises 4,497,443 contigs covering approximately 2.1 Gb of unmasked sequences. For SNPs located in the repetitive fraction of the genome, ISBP markers were designed from contigs [20]. A total of 6,816,965 ISBPs were identified of which 1,003,684 were high confidence and 2,837,656 were medium confidence markers. High and medium-confidence ISBPs were subjected to clustering to discard non-unique regions. Eventually, 2,452,835 unique ISBPs covering ~523 Mb were used as a reference sequence for ISBP-derived SNP discovery.

SNP calling and filtering predicted 3,289,847 intervarietal SNPs (as opposed to intravarietal SNPs present between homoeologous loci), of which 1,231,106 (37%) were specific to European lines, 1,117,588 (34%) specific to Asian lines and 941,153 (29%) shared between both pools. SNPs were compared with those recently described by Montenegro *et al*. [15]. Eventually, 2,693,494 were unambiguously mapped on the wheat genome assembly of which 54% were found to be common between the two sets and 46% were found to be specific to our dataset, which is consistent with the 37% European-specific polymorphism rate observed. Out of the 3.3 million SNPs, 2,336,545 were located in low-copy sequences and 953,302 in ISBPs (Table 1 and S1 Table at https://figshare.com/articles/Supplemental_Table_S1_zip/5501329). Strong differences were observed in terms of SNP proportion between homoeologous genomes, with the B-genome being the more polymorphic (1,607,282 SNPs; 49%), followed by the A-genome (1,357,405; 41%) then the D-genome (325,160; 10%). Within-subgenome differences were also observed. For example, in the B-genome, SNP density ranges from 125 SNPs / Mb on 4B to 334 SNPs / Mb on 3B. Finally, the SNP density was also found to vary greatly between homoeologous groups, the group 4 representing only 8% of the whole SNPs, whereas the other groups represent 15% on average. In addition to a reduced diversity level, the D-genome also exhibits a higher percentage of private SNPs, *i*.*e*. polymorphisms observed in only one line (on average, 29% for the D-genome *vs*. 21% for the A- and B-genomes).

**Table 1:** Summary of the SNP number.

	ISBP-derived	Intergenic	Exonic	Intronic	Total
A-genome	390,462	866,599	38,485	61,859	1,357,405
1A	60,774	131,481	5,851	9,367	207,473
2A	75,889	153,026	7,058	11,423	247,396
3A	40,179	98,901	4,433	7,028	150,541
4A	36,467	90,892	4,464	6,241	138,064
5A	56,534	118,432	4,493	8,575	188,034
6A	54,744	113,872	6,429	9,272	184,317
7A	65,875	159,995	5,757	9,953	241,580
B-genome	469,964	1,021,845	44,266	71,207	1,607,282
1B	59,926	136,537	5,517	9,600	211,580
2B	81,977	168,903	9,864	14,169	274,913
3B	96,264	213,332	8,219	13,499	331,314
4B	32,557	62,266	3,055	4,920	102,798
5B	75,310	163,224	8,627	13,169	260,330
6B	58,675	136,036	5,071	8,371	208,153
7B	65,255	141,547	3,913	7,479	218,194
D-genome	92,876	205,378	11,451	15,455	325,160
1D	15,977	38,373	2,271	2,874	59,495
2D	17,933	38,924	2,737	4,257	63,851
3D	9,846	23,035	921	1,280	35,082
4D	8,893	14,271	511	620	24,295
5D	12,343	26,679	1,639	2,079	42,740
6D	11,065	26,582	1,664	2,072	41,383
7D	16,819	37,514	1,708	2,273	58,314
Total	953,302	2,093,822	94,202	148,521	3,289,847

The majority of SNPs (68%) corresponded to transition (Ts) and 32% to transversion (Tv), leading to an average Ts/Tv ratio of 2.12, a value that is similar to what has been reported previously in wheat [9,11,18]. This ratio was found to be higher in ISBPs (2.28) than in low copy regions (2.06), probably as a result of the high methylation level in TEs that leads to an increase in mutation frequency at deaminated sites [33,34].

### Development of a high density SNP genotyping array for wheat

A high-density Affymetrix Axiom genotyping array was designed using a subset of the 3.3 million SNPs. For the selection of SNPs, several criteria were applied. First, only SNPs found in at least two out of the eight lines and polymorphic in European germplasm were selected. Second, the number of SNPs per chromosome arm was chosen according to arm size. However, because of the natural lack of polymorphism in the D-genome, we decided to double our genotyping array in D-genome-originating SNPs. Third, SNP context sequences were aligned against contigs from the chromosome-based draft sequence of the wheat genome [25] and loci having multiple hits in the genome were discarded. Finally, both intergenic and genic SNPs were selected, which departs from other genotyping arrays that mainly focused on genic polymorphisms [9,10,21].

Eventually, our Axiom genotyping array (hereafter referred to as the TaBW280K array) comprised 280,226 SNPs, including 225,596 intergenic and 54,280 genic SNPs (S1 Table). The percentage of SNPs for the A-, B- and D- genome was 39%, 42% and 19%, respectively. Regardless of the genome, the number of SNPs per chromosome arm was highly correlated with the arm size (R>0.9; p<1E-6). The SNP density was one marker every 52 kb on the A-genome, one every 53 kb on the B-genome and one every 92 kb on the D-genome. These 280,226 SNPs originated from 117,799 independent International Wheat Genome Sequencing Consortium (IWGSC) contigs and covered 19,085 predicted genes.

**Fig 1:**
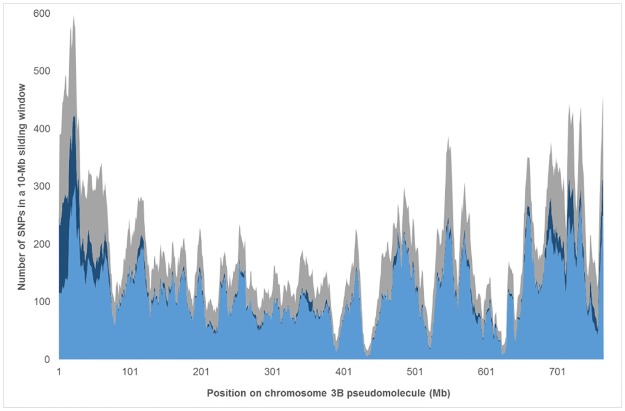
Distribution of the TaBW280K SNPs along chromosome 3B. Densities of Polymorphic High Resolution SNPs (light blue), Off-Target Variants (dark blue) and non-converted (grey) SNPs are computed in a 10-Mb sliding window (step 1 Mb). The X-axis represents the position on chromosome 3B pseudomolecule (in Mb).

Since good coverage of the genome is an important feature of genotyping arrays, we assessed the quality of our coverage by studying physical distribution of selected SNPs along chromosome 3B, the only chromosome for which a reference sequence was publicly available [23]. Out of the 280,226 SNPs, 18,745 were assigned to this chromosome. By aligning SNP context sequences to the 3B pseudomolecule, 15,123 were unambiguously mapped to a single position. The average density was of 192 SNPs / 10 Mb and the median density, 169 SNPs / 10 Mb, ranging from 16 to 592 (Fig 1). Only 7.7% of inter-SNP distances were larger than 200 kb, the largest region without any single SNP being 2.56 Mb-long. Two main regions of low SNP density were observed on the long arm of chromosome 3B (approx. 430–440 Mb and 620–630 Mb).

Performance of the TaBW280K genotyping array was examined by genotyping a set of 96 wheat accessions comprising 13 European elite varieties as well as 83 accessions from a core collection representing the world-wide diversity [35] (S2 Table). SNPs were classified in six main categories according to cluster patterns produced by the Affymetrix software: Polymorphic High Resolution (PHR; 165,885; 59%), Off-Target Variants (OTV; 26,105; 9%), Monomorphic High (MHR; 48,204; 17%), No Minor Homozygous (NMH; 9,370; 4%), Call Rate Below Threshold (CRBT; 16,732; 6%) and Others (13,570; 5%) (Table 2; S1 Table).

**Table 2:** Summary of the number of SNPs per clustering category and clustering metrics.

	Total	Genic	Intergenic
Total	280,226	54,280	225,946
PHR	165,885	38,276	127,609
OTV	26,105	4,182	21,923
MHR	48,204	6,233	41,971
NMH	9,730	361	9,369
CRBT	16,732	2,869	13,863
Other	13,570	2,359	11,211
HomRO > 0.3	148,423	29,807	118,616
HomFLD > 10	100,138	21,873	78,265
Diploidized	92,419	19,158	73,261

**Fig 2:**
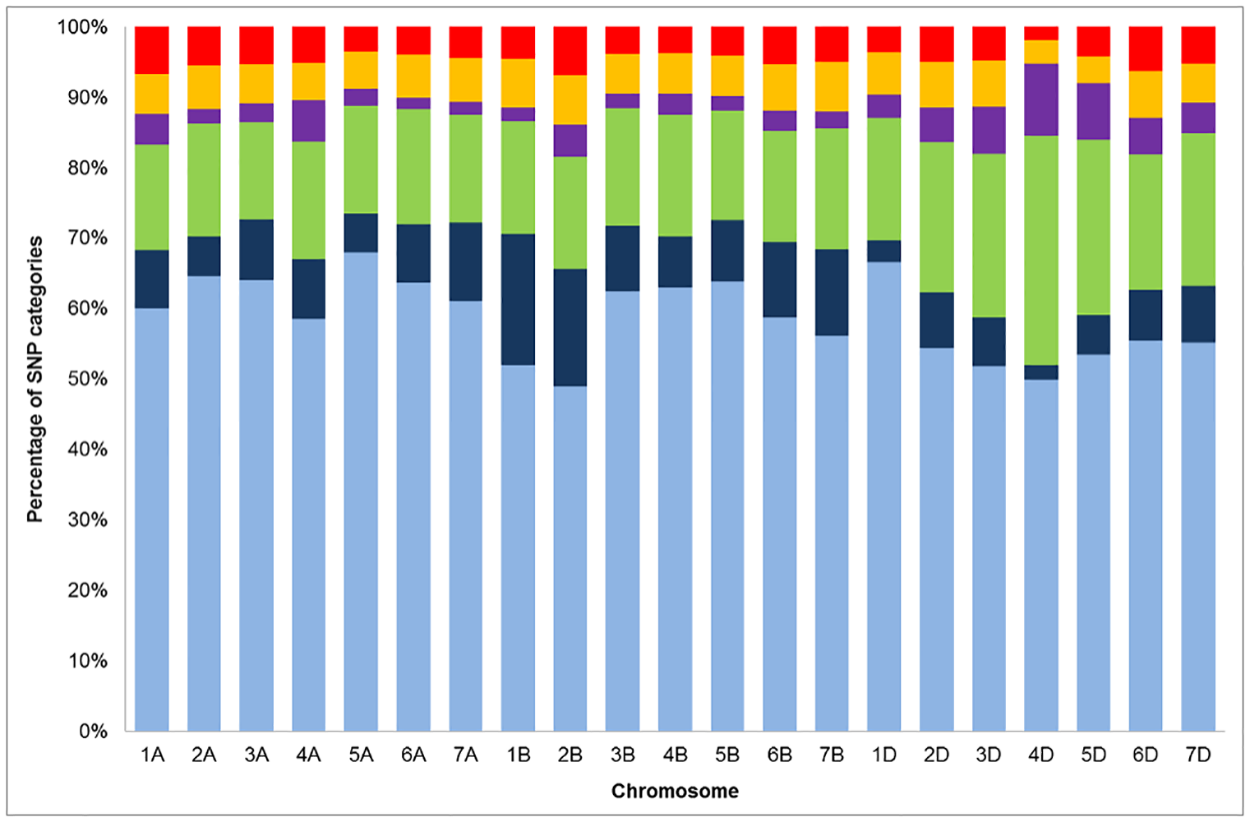
Proportion of TaBW280K SNP clustering categories. For each chromosomes, the percentage of each category is displayed: PHR (light blue), OTV (dark blue), MHR (green), NMH (purple), CRBT (yellow) and Others (red).

Differences were observed between chromosomes (Fig 2). For example, chromosomes 1B and 2B displayed the highest proportion of OTVs, while 4D had the highest proportion on NMH. Differences were also observed between homoeologous genomes, with the B-genome having more OTVs and the D-genome having more NMH.

For subsequent analyses, only PHR and OTV SNPs were considered as converted SNPs, *i*.*e*. polymorphic SNPs that can be efficiently scored on the array. This represented 191,990 SNPs, thus an overall conversion rate of 69%. This conversion rate differed according to SNPs, with 78% of genic and 66% of intergenic SNPs being converted. The number of converted SNPs per chromosome was highly correlated with the overall number of SNPs present on the chip (R = 0.99; p = 0). The converted SNP density was one marker every 74, 79 and 158 kb on the A-, B- and D-genomes, respectively. The absence of bias in the conversion rate was confirmed at the sequence level on chromosome 3B where the number of converted SNPs in a 10-Mb window was highly correlated with the overall number of SNPs in the same window (R = 0.98; p = 0) (Fig 1).

The overall heterozygosity rate was found to be 1.5%, as expected for homozygous lines with limited residual heterozygosity rate. The percentage of missing data per genotype ranged from 0.4 to 5.1%, with 88% of the lines having less than 2% missing data. This can be extrapolated as a 91% “call rate” over the whole analysis. Finally, the percentage of total genotypes returned across all markers, defined as the “completeness”, was 99%, with 1% missing data.

Two other metrics can be used to check probeset quality on Axiom arrays. The first one is the Homozygote ratio Offset (HomRO) that defines the location in the contrast dimension (X-axis) of the homozygous genotype cluster that is the closest to zero and/or most likely to be misplaced. Positive HomRO values indicate that AA and BB clusters are located on each side of zero. Negative values indicate that both clusters are on the same side of zero. Theoretically, in polyploid species where several subgenomes contribute to allele dosage, HomRO values tend to be low. In tetraploid species, HomRO is expected to be close to 0 and below 0.3 and in hexaploid species, below 0 [36]. In our experiment, 148,423 probesets (77% of converted probesets) had a HomRO > 0.3. The second QC metric is the Homozygote Fisher’s Linear Discriminant (HomFLD) which is a measurement of the cluster quality of a SNP. HomFLD examines the distance between the two homozygous clusters as well as the variance across clusters. High FLD values indicate high-quality clusters with well-separated centers and narrow distribution. Classically, for polyploid species, Affymetrix recommends a HomFLD value greater than 3.6. By manually examining SNP clusters, we found that a HomFLD value greater than 10 was a good, yet very conservative, indicator of high quality clusters. In our dataset, 100,138 probesets (52%) had HomFLD values greater than 10. By combining diploid-like HomRO (>0.3) and high HomFLD (>10) values, we identified a set of 92,419 SNPs (48%) that can be considered as diploidized (see S1 Fig for examples). The percentage of diploidized SNPs slightly differs between genic and intergenic, with 45% and 49%, respectively. While the percentage of probesets having a HomFLD > 10 is similar for both types of SNPs, the main difference comes from the polyploidized SNPs (HomRO < 0.3), 30% of genic SNPs and 21% of intergenic SNPs.

The average Polymorphism Index Content among the 96 lines was 0.36, corresponding to an approximate 25 *vs*. 75% distribution. This value is comparable to previously reported PIC values in wheat [17, 18]. It is worth noting that 50% of the SNPs had a PIC value between 0.4 and 0.5 and only 3,165 (2%) were found in one single genotype.

As suggested by Didion *et al*. [37], OTVs can be used to detect presence / absence variations (PAVs). Indeed, they correspond to probes showing four clusters, one of them corresponding to a null allele. In our experiment, 26,105 SNPs were classified as OTVs. The average percentage of converted SNPs corresponding to OTVs per line was 1.5%, ranging from 0.1% for CS to 2.7% for Equinox. In the top ten lines showing the highest OTV rate (>2.0%) were eight lines in which more than 40% of the OTVs were located on the short arm of chromosome 1B (1BS), while the average rate on 1BS for the whole panel was 0.7%. At least six out of these eight lines are known to carry the 1RS.1BL translocation. Another line was BlueBoy in which almost 40% of OTVs were located on chromosome 2B (*vs*. 11.4% for the other lines on average). Finally, not surprisingly, the last one of the top 10 lines was W7984, a synthetic wheat formed by hybridizing a tetraploid wheat *Triticum turgidum* L. subsp. *durum* var ‘Altar 84’ (AABB genotype) with the diploid goat grass *Ae*. *tauschii* (219; CIGM86.940) (DD genotype) [38]. On chromosome 3B, OTV were mainly found in the fast-evolving distal R1 and R3 regions identified by Choulet *et al*. [23] (Fig 1).

The PHR and OTV probesets from the TaBW280K array were compared to that of two other publicly available Axiom chips, namely the high-density wheat 820K array [9] and the breeder-oriented 35K array [22]. 2,600 probesets were found to be shared with the former, which represents 1.4% of the TaBW280K and 0.3% of the 820K. On 3B, the shared SNPs were distributed throughout the entire chromosome, with an average distance of 4.4 Mb (median = 2.7 Mb). For the breeder-oriented chip, 949 probesets were shared, *i*.*e*. 0.5 and 2.7% of the TaBW280K and the 35K arrays, respectively. In both cases, the number of shared SNPs was correlated with the total number of converted SNPs per chromosome (R = 0.84 and R = 0.89, respectively).

### Phylogenetic analysis of wheat lines

**Fig 3:**
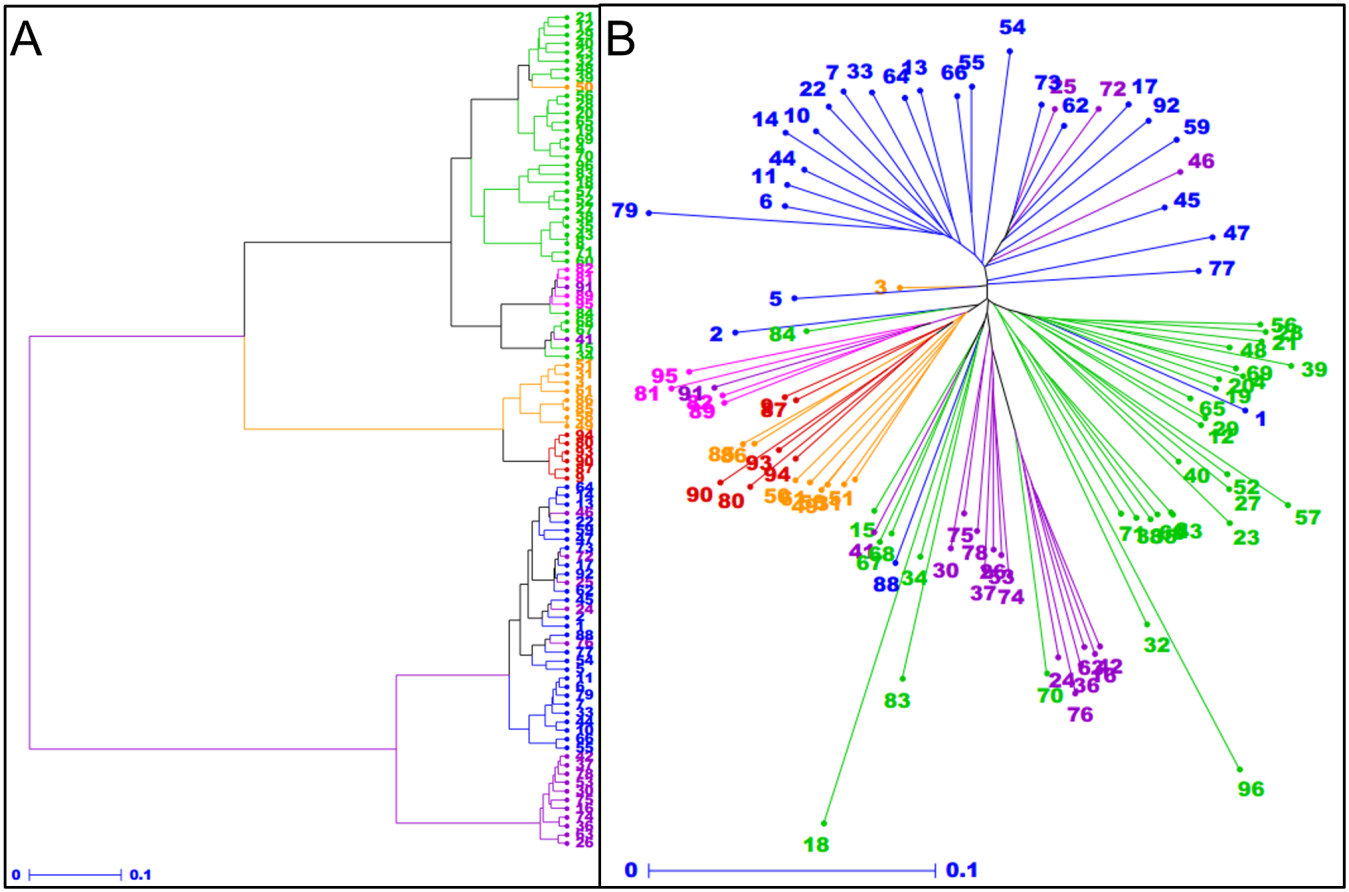
Phylogenetic relationships between 96 wheat accessions. (A) Ward dendrogram showing phylogenetic relationships between wheat accessions revealed by PHR and OTV SNPs. (B) Neighbour-joining tree showing phylogenetic relationships revealed by OTV SNPs only. Accessions are numbered following the S2 Table and colored according to their geographical origins: Eastern Asia (orange), Caucasus (pink), Central Asia (red), Western Europe (blue), Eastern Europe (purple) and Mediterranean (green).

Genotyping data of the 96 wheat accessions with 191,900 PHR and OTV SNPs were used to describe diversity within the panel. Fig 3A illustrates the Ward dendrogram of the 96 wheat accessions. In a previous study on a 367-individual core collection capturing 98% of the worldwide diversity, Horvath *et al*. [39] performed a population structure analysis that led to five groups of accessions which can be related to their geographical origins: Western Europe (WE cluster), Eastern Europe (EE), Mediterranean (Med), Asia (AS) and Nepal (NP). In the present study, the AS cluster was split into three main groups. The first group corresponds to Eastern Asian lines from Japan, China, Korea and India. It is worth noting that the NP group was found to be included in this cluster, close to Indian lines. A second group includes accessions from Turkmenistan, Afghanistan, Tajikistan, Pakistan and Armenia, roughly corresponding to the Central Asia. The third group comprises lines from Turkey, Georgy, Azerbaijan and Russia and could be considered as the Caucasian group. This group was closer from Med cluster than from the AS one. Some lines from Canada, Finland, Algeria and Greece were separated from the Med cluster and grouped together, close to the Caucasian group. Finally, most of the elite varieties were clustered together.

In addition, a set of 5,486 OTVs without any single missing data was used to construct a PAV-based phylogenetic tree (Fig 3B). The geographical groups identified with the whole set of converted SNPs was largely recovered, suggesting that structural variations of the wheat genome, and more precisely PAVs, reflect geographical origins of lines. However it is worth noting that the different geographical groups were not clustered in the same way. Indeed, while the Caucasian cluster was found to be close to the Med one, it appears more closely related to the Eastern and Central Asian clusters when considering only OTVs. Also, the EE lines were clustered with the Med and Asia groups. Finally, five out of the six lines harboring the 1RS.1BL translocation mentioned previously were found to cluster together whereas they were dispersed along the whole tree when considering all PHR and OTV markers.

### Construction of a high-density SNP genetic mapping

The TaBW280K SNP array was used to genotype 430 Single Seed Descent (SSD) individuals derived from a cross between Chinese Spring and Renan (CsRe) [23]. Out of the 280,226 probesets, 85,276 were found to be polymorphic between the two parental lines and PHR on the population. Eventually, 83,721 (98.2%) SNPs were genetically mapped in 21 linkage groups corresponding to the 21 chromosomes of bread wheat, with no unlinked markers (Table 3; S3 Table). The D-genome was the less covered with 18% of the mapped markers while the A- and B-genomes were similarly covered with 41% of mapped markers. The most populated chromosome was 3B (5,811) and the least populated, chromosome 4D (1,507). Overall, the number of mapped SNPs was found to be highly correlated with the total number of PHR markers per chromosome (R = 0.99, p = 0).

**Table 3:** Description of the TaBW280K-based Chinese Spring x Renan genetic map.

	Number of SNPs	Map length (cM)	Number of unique genetic positions	Number of non-redundant SNPs	Bin length (cM)
A-genome	34,646	1,088	2,434	809	0.45
1A	5,294	147	423	120	0.35
2A	5,870	107	290	93	0.37
3A	5,019	156	343	121	0.45
4A	4,933	181	294	119	0.62
5A	4,194	193	423	138	0.46
6A	4,025	127	237	87	0.54
7A	5,311	176	424	131	0.42
B-genome	34,242	958	2,331	745	0.41
1B	5,523	133	375	106	0.35
2B	4,283	131	307	85	0.43
3B	5,811	164	412	127	0.40
4B	3,489	113	262	104	0.43
5B	4,748	178	361	124	0.49
6B	5,932	113	298	95	0.38
7B	4,456	126	316	104	0.40
D-genome	14,833	1,261	2,004	1,062	0.63
1D	2,091	158	225	124	0.70
2D	2,468	151	235	119	0.64
3D	2,436	200	333	173	0.60
4D	1,507	149	311	164	0.48
5D	1,710	220	324	190	0.68
6D	2,464	185	256	132	0.72
7D	2,157	198	320	160	0.62
Total	83,721	3,308	6,769	2,616	0.49

The genetic map covers 3,308 cM. The D-chromosomes had the longest genetic maps, with an average of 180 cM and a cumulative length of 1,261 cM, followed by the A-chromosomes (mean length = 155 cM; cumulative length = 1,088 cM) and the B-chromosomes (mean = 137 cM; cumulative = 958 cM).

Out of the 83,721 SNPs, 81,105 (97%) cosegregated with at least another marker, while 2,616 corresponded to non-redundant loci. The proportion of non-redundant SNPs was higher on the D-genome (7%) than on the A- and B-genomes (2%), as a result of the lower number of markers and higher recombination rate. The number of unique genetic positions was 6,769 and the average size of the corresponding genetic bins was 0.49 cM, ranging from 0.35 to 0.72 cM, for chromosomes 1A and 6D, respectively. Sixty-four percent of genetic bins were smaller than 0.3 cM and less than 1% larger than 5 cM. The three largest gaps were located on chromosomes 6D (25.4 cM), 7A (12.3 cM) and 5D (9.4 cM).

The quality of our genetic map was checked by aligning it to the POPSEQ map [12]. The average correlation between the two genetic maps was found to be very high (R = 0.94; p = 0.) (S2 Fig). A drop in correlation was observed on chromosome 4D (R = 0.52) that is caused by a large gap between 20 and 60 cM in the POPSEQ map. As this map has been constructed with a synthetic wheat, one can hypothesize that the *Aegilops tauschii* chromosome 4D differs from that of bread wheat. A very good correlation was also observed between our map and the Wheat660K SNP array-derived KN9204xJ411 genetic map (R = 0.97; p = 0) [40]. Major discrepancies were found on chromosomes 1BS; 5BS and 7DL that can be explained by the structure of the KN9204 and J411 genomes. Indeed, KN9204 carries the 1RS/1BL translocation resulting in distorted segregation of 1BS markers. In addition, no polymorphic markers were found on 5BS in this cross thus this chromosome arm was not included in the genetic map. Finally, a segment inversion was identified on chromosome 7DL that is not present in our genetic map.

## Discussion

By combining a draft assembly of the hexaploid wheat genome [25] with whole-genome resequencing data, we conducted a genome-wide SNP discovery. To this aim, eight wheat accessions were selected of which four belong to the European genepool and four to the Asian genepool (Australia, Israel and China). Indeed, as worldwide wheat diversity has been shown to be clearly divided according to wheat’s European and Asian origins, this selection allowed for a well-balanced design for SNP discovery [35]. Eventually, almost 3.3 million polymorphisms were identified at the whole genome level. This resource offers the possibility to investigate not only genic but also intergenic regions, including the repetitive fraction that has been shown to be a great source of SNPs for wheat genetics and breeding [18,20]. The overall proportions and distribution of SNPs between homoeologous genomes and chromosomes were quite consistent with previous studies in wheat [10,11,14,41]. However, the proportion of D-genome SNPs identified in our study was slightly lower than previously reported (10% *vs*. ~17%, respectively). This is likely due to the limited genetic diversity present in our SNP discovery panel. Consistent with that is the fact that by sequencing a set of 43 bread wheat accessions and wheat relatives, Winfield *et al*. [9] found an almost identical SNP proportion between the three homoeologous genomes. The higher proportion of private SNPs in the D-genome reported in this study as well as by Jordan *et al*. [10] also points towards this explanation and is consistent with the hypothesis of a limited number of ancestral D-genome donors involved in the hexaploidization process [42].

These SNPs were used to design a high-throughput Axiom SNP array comprising 280,226 SNPs. Several other genotyping arrays have already been designed, such as the Illumina iSelect 90K SNP array [11], the Affymetrix Axiom Wheat660 [40] and the Affymetrix Axiom 820K [9]. Ours departs from the others in several regards. First, it has a higher density compared to the former while staying in a one-array format compared to the latter that requires two arrays. Second, by contrast with other genotyping chips already available that focused on genic SNPs, we chose to incorporate both intergenic (81%) and genic (19%) polymorphisms, therefore allowing us to cover efficiently both gene-rich and gene-poor regions. As a result, even though the converted SNP density was not even along chromosomes, few megabase-sized regions without SNPs were observed. Finally, while the overall conversion rates between the different genotyping arrays were found to be similar, several differences can be observed. In the present study, the percentage of converted SNPs was 69% with 59% being PHRs and 9% being OTVs. While a similar percentage was reported by Winfield *et al*. [9] on the genotyping of 475 accessions with the Axiom 820K SNP array (67%), only 7% were PHRs, the vast majority being NMHs (55%). Similarly, out of the 81,587 SNPs present on the Illumina iSelect 90K SNP array, 56,388 (69%) were considered as converted. However, only 35,684 (44%) showed three distinct clusters, of which 20,785 (25%) had well-separated clusters that were correctly captured by the default algorithm [11]. By contrast, no manual curation of clustering results was required for our genotyping data. The low NMH rate observed in our study (4%), together with the high percentage of diploidized converted SNPs (48%) demonstrates the efficacy of our SNP selection procedure that excluded multiple-hits loci, therefore resulting in unique probesets. In addition, the higher amount of intergenic diploidized SNPs compared to genic ones strongly suggests that using non-repetitive intergenic region-derived markers is an efficient way to design pseudodiploid genotyping arrays. The genome-specificity underlying this pseudodiploid behavior might also be a key factor for the efficient scoring of PAVs. Indeed, while our panel was much narrower from a genetic viewpoint than the one used by Winfield and collaborators, our array detected a much higher proportion of OTVs (9% *vs*. 5%, respectively). One might also consider that data originating from diploidized SNPs would allow for copy number variation analyses, as demonstrated in cattle [43] and human [44,45].

It is worth noting that 2,600 and 949 SNPs were shared with the 820K [9] and the 35K Axiom [22] arrays, respectively. The higher percentage of SNPs shared with the 35K (2.7%) compared to the 820K (0.3%) is expected since both the TaBW280K and the 35K were enriched in polymorphisms present in the European elite material whereas the 820K contained a high proportion of wheat relative SNPs.

Being able to score PAVs and CNVs is particularly relevant, considering the growing body of evidences of the role of structural variations in the expression of phenotypes not only in human [46,47] but also in plants [48–52]. Here, we showed that PAVs were mainly located in the distal regions of chromosome 3B, which have been described as the fast-evolving recombinogenic chromosomal parts that are enriched in nonsyntenic genes as well as in genes differentially expressed and potentially involved in wheat adaptation [24,53]. In addition, our PAV-based phylogenetic analysis, while recovering the classical geographical clustering of wheat lines, also revealed differences that might reflect a different type of diversity and evolution. Taken together, these results are of particular interest as adaptation has been associated with intraspecific structural variations [54]. A detailed analysis of chromosome segments showing differential structural variations between populations might therefore provide with an opportunity to identify genetic factors involved in adaptive traits to specific environmental factors.

The TaBW280K SNP array was proven to be an efficient tool to characterize genetic resources. While clustering of accessions was mainly consistent with a previously reported structure [39], the AS cluster was split in three (Eastern Asia, Central Asia and Caucasus) that are highly relevant from a geographical viewpoint. Also, even though elite varieties were clustered together as a result of the narrow genetic diversity found in the European germplasm, they were well separated in the phylogenetic tree, therefore reinforcing the idea that the TaBW280K is a powerful tool to discriminate between closely related lines. Indeed, 91% of converted SNPs (174,360) were found to be polymorphic in European elite material. This proportion is significantly higher than the one observed on the Affymetrix Axiom 820K array (18%) that was primarily designed to characterize a wide range of wheat accessions and relatives, as well as on the Illumina iSelect 90K array (37%). It is also worth noting that our array was also proven to be efficient to genotype tetraploid wheats, with 72% of polymorphic SNPs *vs*. 11% and 36% for the Affymetrix Axiom 820K and Illumina iSelect 90K arrays, respectively (Jacques Davis, personal communication). So far, the TaBW280K has been used by our group to genotype more than 7,800 wheat accessions including 4,600 covering the worldwide genetic diversity as well as elite varieties and breeding material to conduct genome-wide association studies and implement genomic selection models (unpublished data). As it is publicly available, it has also been purchased by other groups in the world.

Finally, our SNP array was used to construct an 83K SNP genetic map. All chromosomes were densely populated, with an average of 3,987 SNPs per chromosome, ranging from 1,507 to 5,932. The vast majority (97%) of SNPs was found to cosegregate with at least one other marker. This is likely to be due to the high number of markers relative to the small number of COs per meiosis observed in plants, as well as to the relatively small size of the mapping population [55,56]. In addition, it is well known that a significant portion of wheat chromosomes are recombination-poor, leading even distant loci to cosegregate [23,57]. Even though the D-genome contains fewer markers than the two others, it displays a higher recombination rate. Such a negative relationship between the polymorphism level and the recombination rate has already been reported in wheat and strongly suggests an impact of the sequence divergence on the occurrence of crossing-overs [58]. In the recent years, thanks to the advent of high-throughput genotyping approaches, several high density genetic maps have been produced. For example, Wang *et al*. [11] used a combination of eight doubled-haploid mapping populations to order 46,977 SNPs with the Illumina iSelect 90K SNP array. Using the same SNP array, an 18,601-SNP genetic map was constructed from an eight-parent MAGIC population [59]. Winfield *et al*. [9] generated a consensus of three different genetic maps (Avalon x Cadenza, Savannah x Rialto and W7984 x Opata) using the Axiom 820K SNP array. Cui *et al*. [40] constructed a 119,566 loci high-density genetic map by genotyping 188 RIL lines derived from a cross between Kenong 9204 (KN9204) and Jing 411 (J411) with the Axiom Wheat660K SNP array. Chapman *et al*. [60] used POPseq to construct a 113K marker-map of the W7984 x Opata population. While it comprises a number of markers that is quite comparable to other genetic maps, our map benefits from two main advantages. First, it has been generated from a single biparental cross, therefore reducing the impact of potential structural genomic variations between parents, which is a challenging problem in consensus maps [60]. Second, our map is a high-density genetic map generated from a cross involving Chinese Spring, the cultivar that has been selected by the wheat community as the reference to produce a high quality reference sequence. Other biparental crosses involving Chinese Spring have been used to construct genetic maps but none of the publicly available ones has the same SNP density. For example, the genetic map generated by Gao *et al*. [61] and Wen *et al*. [62] used a Zhou 8425B × Chinese Spring population to map 5,636 and 14,955 markers, respectively. For these reasons, our map has been selected by the IWGSC as the reference genetic map to anchor and order the wheat genome reference sequence (unpublished data). In this context, it has also been used to genotype a radiation hybrid panel aiming at validating the IWGSC Reference Sequence assembly v1.0 (Vijay Kumar, personal communication).

In conclusion, the large collection of SNPs as well as the high-throughput genotyping array and the dense genetic map described here provide the wheat community with new tools for genetic and genomic studies, as well as for marker-assisted breeding. With the annotated reference sequence of the wheat genome to come in a few months, polymorphisms will be ordered along chromosomes, therefore allowing for a more precise characterization of haplotypes, a better definition of introgression boundaries and a faster map-based cloning of genes underlying traits. In addition, a lower density 35K SNP array derived from the TaBW280K chip is currently being developed to allow for cost-efficient genotyping of large populations.

## Supporting information

S1 Table The TaBW280K SNP array. List of 280,226 SNPs with probeset ID, SNP ID, fraction (genic or intergenic), chromosomal origin, corresponding IWGSC contig, context sequence, alleles, SNP category (PHR, OTV, MHR, NMH, CRBT and Other), numbers of AA, AB, BB, OTV and missing data, and PIC value.
(ZIP)

S2 Table List of 96 wheat lines genotyped with the TaBW280K SNP array. (TXT)

S3 Table An 83K loci genetic map of the Chinese Spring x Renan population. (ZIP)

S1 Fig Examples of diploidized (HomRO > 0.3 and high HomFLD > 10) SNPs. A-B: Contrast = log_2_[(#A x 100 +100) / (#B x 100 +100)]; (A+B)/2: Size = [log2(#A x 100 +100) + log2(#B x 100 +100)] / 2.
(PDF)

S2 Fig Comparison between the Chinese Spring X Renan genetic map and the W7984 x Opata POPseq genetic map (Poland *et al*., 2012). Correlations between contig orders from both maps are indicated for each chromosome.
(PDF)
