# Supplementary figures and images for: High throughput SNP discovery and genotyping in hexaploid wheat
Source: PLoS One. 2018 Jan 2;13(1):e0186329. doi: 10.1371/journal.pone.0186329 (PMC5749704; doi:10.1371/journal.pone.0186329)

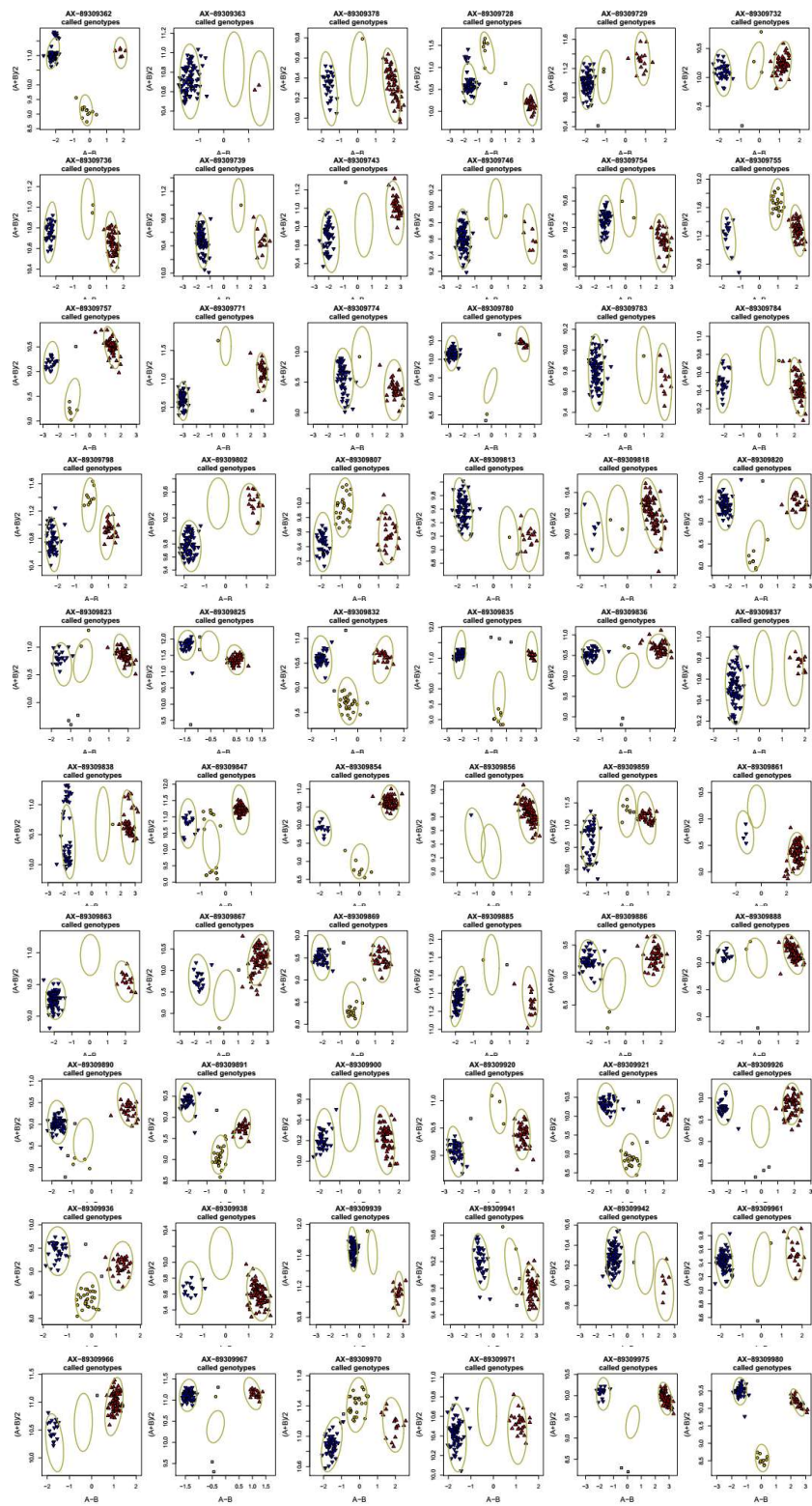

Supplement: S1 Fig — A-B: Contrast = log2[(#A x 100 +100) / (#B x 100 +100)]; (A+B)/2: Size = [log2(#A x 100 +100) + log2(#B x 100 +100)] / 2. (PDF) [file pone.0186329.s004.pdf]

POPseq maps (cM)

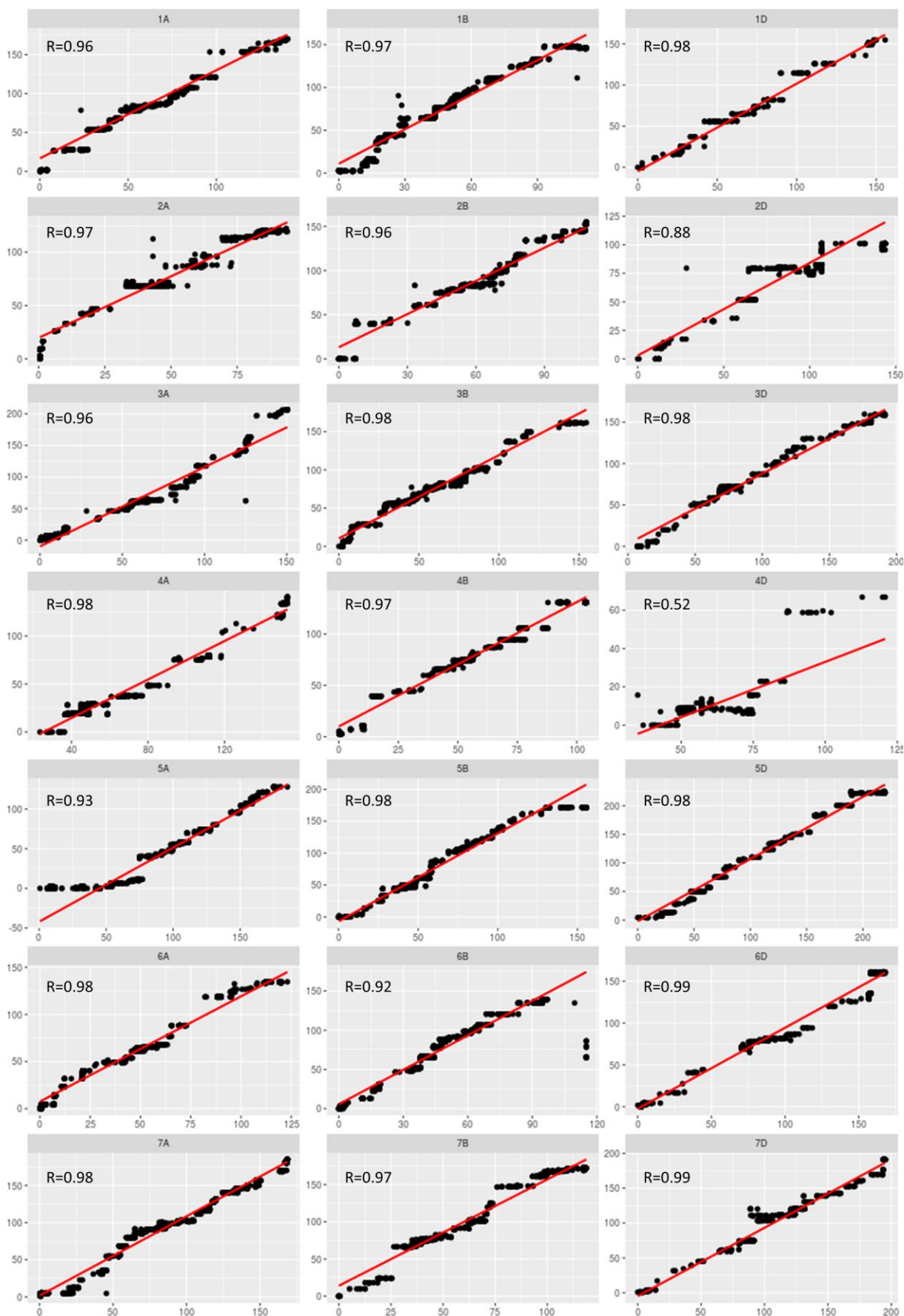

Chinese Spring x Renan maps (cM)

Supplement: S2 Fig — Correlations between contig orders from both maps are indicated for each chromosome. (PDF) [file pone.0186329.s005.pdf]
